# Transcriptome, proteome and functional characterization reveals salt stress tolerance mechanisms in upland cotton (*Gossypium hirsutum* L.)

**DOI:** 10.3389/fpls.2023.1092616

**Published:** 2023-02-16

**Authors:** Kangtai Sun, Teame Gereziher Mehari, Hui Fang, Jinlei Han, Xuehan Huo, Jingxia Zhang, Yu Chen, Dongmei Wang, Zhimin Zhuang, Allah Ditta, Muhammad K.R. Khan, Jun Zhang, Kai Wang, Baohua Wang

**Affiliations:** ^1^ School of Life Sciences, Nantong University, Nantong, Jiangsu, China; ^2^ State Key Laboratory of Cotton Biology, Institute of Cotton Research of Chinese Academy of Agricultural Sciences, Anyang, Henan, China; ^3^ Key Laboratory of Cotton Breeding and Cultivation in Huang-Huai-Hai Plain, Ministry of Agriculture and Rural Affairs of China, Institute of Industrial Crops, Shandong Academy of Agricultural Sciences, Jinan, Shandong, China; ^4^ Plant Breeding and Genetics Division, Nuclear Institute for Agriculture and Biology, Faisalabad, Pakistan

**Keywords:** upland cotton, salt stress, transcriptome, proteome, qRT-PCR, VIGS

## Abstract

Uncovering the underlying mechanism of salt tolerance is important to breed cotton varieties with improved salt tolerance. In this study, transcriptome and proteome sequencing were performed on upland cotton (*Gossypium hirsutum* L.) variety under salt stress, and integrated analysis was carried out to exploit salt-tolerance genes in cotton. Enrichment analysis using Gene Ontology (GO) and Kyoto Encyclopedia of Genes and Genomes (KEGG) was performed on differentially expressed genes (DEGs) obtained from transcriptome and proteome sequencing. GO enrichment was carried out mainly in the cell membrane, organelle, cellular process, metabolic process, and stress response. The expression of 23,981 genes was changed in physiological and biochemical processes such as cell metabolism. The metabolic pathways obtained by KEGG enrichment included glycerolipid metabolism, sesquiterpene and triterpenoid biosynthesis, flavonoid production, and plant hormone signal transduction. Combined transcriptome and proteome analysis to screen and annotate DEGs yielded 24 candidate genes with significant differential expression. The quantitative real-time polymerase chain reaction (qRT-PCR) validation of the candidate genes showed that two genes (*Gh_D11G0978* and *Gh_D10G0907*) responded significantly to the induction of NaCl, and these two genes were further selected as target genes for gene cloning and functional validation through virus-induced gene silencing (VIGS). The silenced plants exhibited early wilting with a greater degree of salt damage under salt treatment. Moreover, they showed higher levels of reactive oxygen species (ROS) than the control. Therefore, we can infer that these two genes have a pivotal role in the response to salt stress in upland cotton. The findings in this research will facilitate the breeding of salt tolerance cotton varieties that can be grown on saline alkaline lands.

## Introduction

There are large areas of saline land around the world with complex compositions and varying degrees of salinity, collectively referred to as saline land (Muchate et al., 2016; Abdelraheem et al., 2019). Currently, approximately 20% of the world’s soils are affected by salinization, and the trend is worsening. More than half of the arable land of the world is expected to be salinized by 2050, seriously wasting large areas of land and severely affecting crop production due to the toxicity of ions (Duan et al., 2022; Wang et al., 2022a; Wang et al., 2022b).

Due to the severe salinization of soils by seawater, which is detrimental to plant growth, the cultivation of salt-tolerant crops has become a new direction in modern agricultural development. Cotton, as a salt-tolerant plant, is suitable for growing in saline soils with a salt content under 0.3% (Sun et al., 2018; Sikder et al., 2020), whereas the tolerance level needs to be improved to exploit saline soils. Uncovering the underlying mechanism of salt tolerance is important to breed cotton varieties with improved salt tolerance.

Plants are exposed to biotic and abiotic stresses, such as pathogen infections, pest attacks, extreme temperatures, drought, and salinity (Huang et al., 2019). In response, plants have evolved some sophisticated defense mechanisms, including oxidative burst, the regulation of signaling networks, physiological, molecular, and cellular modifications, to combat these stresses (Khan et al., 2018). Many stress-inducible genes with various functions have been identified in Arabidopsis, rice, and other plants, including a number of transcription factors that regulate stress-inducible gene expression. The products of stress-inducible genes function both in the initial stress response and in establishing plant stress tolerance (Meng et al., 2021).

Transcriptome analysis is a method for studying differential gene expression, functional annotation, metabolic regulation prediction and detection of single nucleotide polymorphism (SNP) loci. Zhang et al. (2016) explored the salt tolerance mechanism of cotton through *Gossypium davidsonii* transcriptome analysis and annotated the signaling pathways for salt overly sensitivity (SOS) and reactive oxygen species (ROS). Guo et al. found several candidate genes for enhancing salt tolerance in upland cotton were (Guo et al., 2015; Guo et al., 2021). Transcriptome sequencing data revealed the presence of the WRKY transcription factor in the salt-tolerant wild cotton species *Gossypium aridum* (Fan et al., 2015). Currently, significant progress has been made in the sequencing of upland cotton genomes (Li et al., 2015; Zhang et al., 2015; Hu et al., 2019; Wang et al., 2019; Yang et al., 2019; Chen et al., 2020; Huang et al., 2020), which provides a basis for the discovery of functional genes and elucidation of key pathways in cotton. The comprehensive application of high-throughput second-generation transcriptome sequencing is conducive to the more rapid identification of candidate genes and exploration of the physiological and metabolic processes of salt-tolerant varieties. GO keywords and KEGG pathways was found to be associated with salt stress regulation, ‘transport’, ‘response to hormonal stimulation’ and ‘signal transduction’ pathways during salt stress played important roles, while protein kinases were implicated in activity and transport pathways (Guo et al., 2015).

Genes involved in hormone biosynthesis and signaling, ROS, and salt overly sensitive (SOS) signaling were discovered by examining the transcriptome of the wild diploid cotton species *Gossypium klotzschianum* for salt stress, indicating their important roles in signaling, redox homeostasis and ion homeostasis in the salinity response (Wei et al., 2017). The results have also revealed differential splicing of genes associated with salt stress, which can contribute to the discovery of candidate genes and the development of molecular markers for salt tolerance breeding in cotton (Zhu et al., 2018).

The genome of an organism is relatively static; although transcription is regulated in different ways, information on regulation or posttranslational modifications cannot be simply obtained by the expression of transcripts (Zhang et al., 2016b). Proteomic analysis, which identifies and quantifies proteins in complex mixtures, has been frequently used during the last few years to uncover the processes in which plants respond to abiotic stresses (Jan et al., 2022). After translation, proteins are usually modified in different ways to function, and some proteins may also be hydrolyzed when they finish their work, so functional gene expression profiling is possible only by proteomic analysis. Quantitative analysis at the protein level is essential to determine the reaction of the plant to salt stress (Zhu et al., 2021).

In this research, integrated transcriptome and proteomics analysis based on the salt-tolerant upland cotton variety ‘Tong Yan No. 1’ under salt stress was performed to uncover the underlying mechanisms of salt tolerance in cotton and identify candidate genes by expression and functional validation. The identification of key genes and important pathways in response to salt stress in cotton will facilitate the discovery of candidate genes and molecular marker development for salt stress tolerance in cotton, which is important for the selection and breeding of salt-tolerant cotton varieties to improve and utilize saline lands.

## Materials and methods

### Plant material and salt treatments

In this investigation, the salt-tolerant upland cotton variety ‘Tong Yan No. 1’ was employed. Plump cotton seeds were selected and disinfected by soaking in dilute hydrochloric acid, and then they were planted in pots of 2.5 L capacity with a 3:1 nutritional to vermiculite soil ratio in a greenhouse at 25°C/20°C and a 16 h/8 h light to dark ratio and watered every three days. Six uniformly growing seedlings at the three-leaf stage were chosen and separated into two groups: the control group and the experimental group. At 5 pm each day, the experimental group was watered with 200 ml of 250 mmol/L NaCl solution, whereas the control group was watered with the same amount of distilled water. After two days, true leaves collected from the three plants in the control group were marked as CK1, CK2 and CK3, whereas true leaves collected from the three plants in the experimental group were marked as Salt1, Salt2 and Salt3. The six samples were frozen on dry ice and sent to Biomics Biotech Co., Ltd. (Beijing, China) for both transcriptome and proteome sequencing.

### Transcriptome sequencing analysis

Total RNA was first extracted from the samples, and its quality was confirmed with agarose gel electrophoresis (AGE). The cDNA libraries were constructed with high-quality RNA (Gubler and Hoffman, 1983). Following the successful completion of the library test, the different libraries were pooled and sequenced using the Illumina HiSeq platform (San Diego, CA, USA). The raw data were filtered to obtain clean data, and the sequences were aligned with the specified reference genome to obtain mapped data for the insert length test (Zhang et al., 2015), randomness test, variable splicing analysis, new gene discovery and gene structure optimization analysis. Differential expression analysis, functional annotation, functional enrichment of DEGs, and other expression level analyses were carried out based on the expression levels of genes in different samples.

The upland cotton TM-1 genome data used in this study were downloaded from the cotton database of Nanjing Agricultural University (https://mascotton.njau.edu.cn/Data.htm) (Zhang et al., 2015).

### Proteome sequencing analysis

Proteins were first extracted from the tissue samples. The concentration of the extracted total protein was determined as described by Bradford (1976). The extracted protein samples were then subjected to reductive alkylation. Then pancreatic enzyme was added with the mass ratio of 1:50 (trypsin: protein) The peptides were labeled with a labeling reagent, and the labeled peptides from each sample were mixed in equal amounts and preseparated using strong cation exchange chromatography. The final peptides were analyzed by liquid phase tandem mass spectrometry.

### Bioinformatics analysis

GO and KEGG analyses of transcriptomic and proteomic DEGs were used to obtain functional annotations related to metabolic pathways. GO analysis determines the function of a gene by analyzing its sequence information. The GOSeq R program was used to perform GO enrichment analysis of differentially expressed proteins (DEPs) and DEGs on the hypergeometric distribution principle (http://geneontology.org/). Further analysis of DEPs and DEGs was carried out using the KEGG database (http://www.genome.jp/kegg/pathway.html). KEGG assays determine the function of genes by analyzing their expression information. The statistical enrichment of DEGs in the KEGG pathway was tested using the KOBAS programmer. The protein-protein interaction networks in candidate genes were analyzed using the Search Tool for the Retrieval of Interacting Genes/Proteins (STRING) Protein Interaction Database (http://string-db.org/). For database screening of DEGs encoding proteins, the data were visualized using cytoscape software (cytoscape 3.6.1).

### Identification and quantitative analysis of candidate gene expression

Sample RNA was extracted using the EASYspin Plus plant total RNA extraction kit from Aidlab (Aidlab Biotechnologies Co., Ltd., Beijing, China). cDNA was synthesized by reverse transcription using M5 Super plus kits purchased from Beijing Mei5 Biotechnology (Mei5 Biotech, Beijing, China). The 2*M5 HIPer Realtime PCR Super mix (SYBR Green) kit (Mei5 Biotech) kit was used for quantitative real-time PCR (qRT-PCR).

Primer5 software was used to design qRT-PCR-specific primers for the candidate genes, using upland cotton *GhUBQ7* (*DQ116441*) as a control for normalization between samples. The sequences of the primers are shown in **Table S1**. qRT-PCR was performed in an ABI 7500 real-time fluorescence quantitative PCR instrument (Applied Biosystems, Signapore), and the relative expression of candidate genes was calculated using 2^-ΔΔCt^ (Livak and Schmittgen, 2001). GraphPad Prism v.9.0.0 software was used to visualize the graphing results.

### Virus induced gene silencing experiment in cotton

Using cDNA of the upland cotton variety ‘Tong Yan No. 1’ as a template, VIGS primers were designed with Snapgene software (Snapgene3.2.1) to select *EcoR I* and *Sca I* enzyme cleavage sites to amplify a 300-500 bp target fragment (Zhang et al., 2017). PCR amplification reactions were performed to obtain the target gene fragment (**Table S2**). The target fragment was recovered using the Tiangen Agarose Gel DNA Recovery Kit (DP209-03) (Tiangen Biotech, Beijing, China) and ligated to the *tobacco rattle virus* (TRV) viral vector (pTRV2) using the enzymatic recombination ligation method (Zhang et al., 2016a). The plasmid containing the VIGS fragment and the helper plasmid were transformed into *Agrobacterium tumefaciens* GV3101 according to the *A. tumefaciens* transformation instructions (Zhang et al., 2020b).

Plants with the silenced *GhCLA1* gene (Cloroplastos alterados 1420 bp) that developed an albino phenotype when silenced were used as a positive control (Zhang et al., 2016a). pTRV:00 was used as the negative control (transformed with the blank pTRV2 vector), whereas pTRV : *Gh_D10G0907* and pTRV : *Gh_D11G0978* were the experimental plants transformed with the *Gh_D10G0907* and *Gh_D11G0978* genes, respectively. The treated plants were transferred to a growth chamber with a temperature of 23°C, a light intensity of 120 μE·m^-2^·S^-1^ and a photoperiod of 16/8 h light/dark. The plants were cultivated for approximately 10 days until the cotton appeared phenotypically.

### Determination of phenotypic and physiological indicators

After 10 days of transfection, *GhCLA1* was silenced in the positive control, and bleaching of the true leaves occurred. To ensure that the target gene was silenced in the experimental group, total RNA was extracted from the negative control and positive control tissues for quantitative analysis using the negative control plants as a reference.

As leaves were needed for the qRT-PCR experiment and the ROS assay, all leaves of the silenced plants were removed from the biomass measurements, leaving only the roots and stems intact. The dry weight and fresh weight of the roots and stems of the plants were measured. Biomass ratio of the plant = dry weight of the plant/fresh weight of the plant (Zhang et al., 2020a).

ROS assays were also performed on silenced cotton plants. In total, 12 plants each of the negative control, pTRV : *Gh_D10G0907*, and pTRV : *Gh_D11G0978* were divided into three replicates, with four leaves in each set of replicates. These plants were also used for subsequent index measurements.

To investigate whether the target gene was associated with salt stress, 200 ml of a 250 mmol/L salt solution was used to water the negative control, positive control, and experimental groups, and phenotypic and physiological measurements were performed after 7 days of treatment. Leaves were collected for ROS content determination using MCE’s H2DCFDA Reactive Oxygen Probe Reagent (MCE, Dallas, TX, USA). The fresh weight of the root and stem of the cotton plant was measured and then baked in an oven at 65°C for 2 days to measure the dry weight. The H2DCFDA Reactive Oxygen Probe Reagent was used according to the kit instructions (https://www.medchemexpress.cn).

### Statistical methods

Statistical analysis and graphing were performed using SPSS v.25.0 and GraphPad Prism v.9.0. A significant difference was declared at the *P* ≤ 0.05 probability level. Three biological replications were maintained throughout the experiment.

## Results

### Transcriptome analysis of salt stress in upland cotton

A total of 10,967 DEGs with |fold-change|> 1 and false discovery rate (FDR) < 0.05, including 7,268 upregulated and 3,699 downregulated genes, were identified. Most of the DEGs were enriched in the metabolism and cell membrane category and the organelle category (**Figure S1**). Under salt stress, both the osmotic changes in plants and the pressure on cell membranes increase, and it is more difficult to transport substances in plants; organelles suffer from water loss and damage from ROS, whereas changes in the internal environment lead to metabolic abnormalities and physiological and biochemical dysfunctions (Sun et al., 2019). Differential GO terms were enriched in cell wall components, microtubules with motor protein and polysaccharide production (**Figure S2**). Under salt stress, plant cells are disrupted by osmotic pressure and rely on the production of large amounts of sugars, synthesized as microtubules and membrane lipids, to maintain normal cell morphology from severe damage. Some of the genes are also related to hormones and antioxidants such as anthocyanins, which reduce the toxicity of ROS to the cells when they are damaged, which is consistent with known studies that have identified plant cellular resilience systems (Roy et al., 2014).

The enrichment factor, q-value, and number of genes enriched in the pathway were used to calculate KEGG enrichment. The top 20 KEGG pathways were selected for display (**Figure S3**). The most significant enrichment of DEGs by KEGG in this study was observed in sesquiterpene and triterpene biosynthesis and glycerophospholipid metabolism (**Figure S3**). Sesquiterpenes and triterpenes are precursors to numerous phytohormones and important metabolites, such as lipids, abscisic acid, and farnesyl pyrophosphate (FPP), all of which have important roles in plant stress resistance. Some of the genes enriched in glycerol ester synthesis were thought to control membrane lipid synthesis, maintain the morphology of the cell membrane and keep the interior of the cell undamaged under adverse conditions (Jiang et al., 2019).

### Proteomic analysis of salt stress in upland cotton

Based on the quantitative results of the proteome, differential expression analysis was performed. Proteins with |fold-change| > 1.5 and FDR < 0.05 were considered proteins with significant differential expression. The results of the salt-stressed proteome revealed 189 DEPs, of which 113 were upregulated, and 76 were downregulated.

The GO analysis showed that DEPs in molecular function were enriched mainly in the entries for membranes, organelles, binding, cellular processes, metabolic processes, and stress responses (**Figure S4**). Salt stress results in many molecular changes in plants (Guo et al., 2022). Among the cellular components, the entries for cells, cell parts, organs and membranes were enriched with more DEPs, indicating that salt stress triggered dynamic regulation of cellular components. The cell membrane metabolic processes and stress responses were significantly affected by salt stress, suggesting that salt stress leads to metabolic dysfunctions such as impaired transport of substances, changes in osmotic pressure, disturbances in cell organization and changes in the physiological and biochemical environment. Although these DEPs respond to salt stress in different ways and may have different functions, they all have an important role in salt stress tolerance in cotton.

GO annotation shows that DEP is enriched in the terms of redox processes, catabolic processes of glucose, chloroplasts and cell wall macromolecules (**Figure S5**). The first effect of high salt stress is the destruction of cell walls and cell membranes, and the synthesis of proteins associated with the cell wall membrane is upregulated to maintain morphology and ensure normal cell growth (Shen et al., 2015). In addition, salt stress disrupts chloroplasts and mitochondria, leading to a reduction in photosynthesis and the breakdown of large amounts of glucose required for normal growth and development. Salt stress leads to an imbalance in the ion content of the cell, and many redox reactions are affected (Guo et al., 2022).

The DEPs were annotated in the KEGG database, and 185 differentially expressed proteins (DEPs) were annotated on 78 pathways after enrichment calculations. The 15 pathway entries with the most significant enrichment were selected for presentation (**Figure S6**). The pathways that were more enriched, such as terpene synthesis, apoptosis, glutathione metabolism, fatty acid degradation and glycerophospholipid metabolism, were all closely related to cell membrane synthesis. Glutathione (GSH) and peroxisomes play an important role as antioxidant systems in plants under stress conditions (Cheng et al., 2023). Initiating apoptosis as a means for plants to protect themselves under stress.

### Integrated transcriptomic and proteomic analysis under salt stress in upland cotton

A total of 24 common DEGs were obtained by integrated transcriptomic and proteomic analysis (**Table 1**), and they were significantly differentially expressed in both the transcriptomic and proteomic results. These 24 genes were selected as candidate genes to study the response mechanism of upland cotton under salt stress.

**Table 1:** Candidate gene names and their expression profiles.

Candidate genes	Homologous genes in *Arabidopsis*	Expression under salt stress
*Gh_D11G0978*	*LEA14-A*	up
*Gh_D11G1883*	*GGPS*	down
*Gh_D07G1329*	*ARP1*	up
*Gh_D10G1253*	*/*	down
*Gh_A01G1685*	*SDC*	up
*Gh_D04G2024*	*/*	down
*Gh_A10G1567*	*/*	up
*Gh_A01G1950*	*SQD2*	down
*Gh_A11G2498*	*RH3*	down
*Gh_A09G1400*	*/*	down
*Gh_D03G0016*	*Stxbp5*	up
*Gh_D11G0441*	*ALDH10A8*	up
*Gh_A05G1463*	*3-Apr*	down
*Gh_A07G2133*	*GDPD2*	down
*Gh_A09G1053*	*COMT*	up
*Gh_A13G0408*	*ISPH*	up
*Gh_D07G0910*	*CYP82G1*	up
*Gh_D03G0971*	*At1g67750*	up
*Gh_D10G0907*	*GGPS*	up
*Gh_D10G1214*	*EP3*	up
*Gh_D09G1404*	*/*	down
*Gh_D13G0457*	*ISPH*	up
*Gh_A10G0282*	*CHLI*	down
*Gh_A05G0722*	*SODCP*	up

Through the preliminary VIGS experiment, we obtained two genes with significant effects. Two significant DEGs, *Gh_D11G0978* and *Gh_D10G0907*, were obtained by summarizing the annotation and function of the target genes and comparing the differential expression fold under salt stress and the control, and the homologous genes were found to be significantly salt tolerant in other species. *The Gh_D11G0978* homolog *LEA* is involved in the regulation of embryonic development under osmotic stress and is found in a variety of crops including maize and wheat (Jia et al., 2014; Miyazaki et al., 2021). *Gh_D10G0907*, a GGPS family gene encoding glycerol-glucoside phosphate synthase that synthesizes glycerol-3-phosphate and adenosine diphosphate (ADP)-glucose into glycerol-glucoside phosphate, is a known salt-tolerant gene in algae and microorganisms (Takashima et al., 2020; Klähn et al., 2021).

### Expression profiles of candidate genes under salt stress

**Figure 1:**
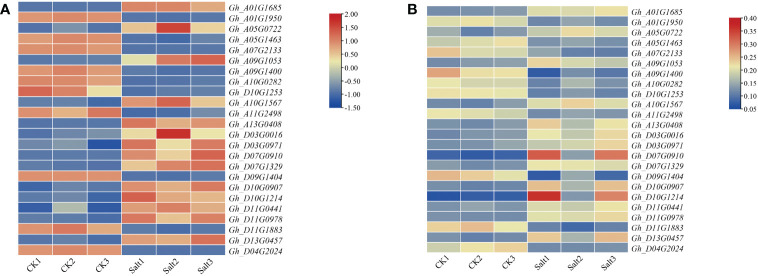
Expression profiles of candidate genes and proteins under salt stress. **(A)** Expression profiles of candidate genes under salt stress. **(B)** Expression profiles of proteins encoded by candidate genes under salt stress. The abscissa in the figure is the sample name, the ordinate is the gene name, and different colors represent different expression values.

Transcriptomic and proteomic expression data of upland cotton under salt stress were extracted to explore the expression pattern of DEGs under salt stress (**Figure 1**). The results of gene expression analysis suggested that the candidate genes have different expression trends in response to abiotic stresses. Among the 24 candidate genes, 14 genes were significantly upregulated in transcription and proteins after salt stress, and 10 genes were significantly downregulated after salt stress.

### Candidate protein interaction analysis

**Figure 2:**
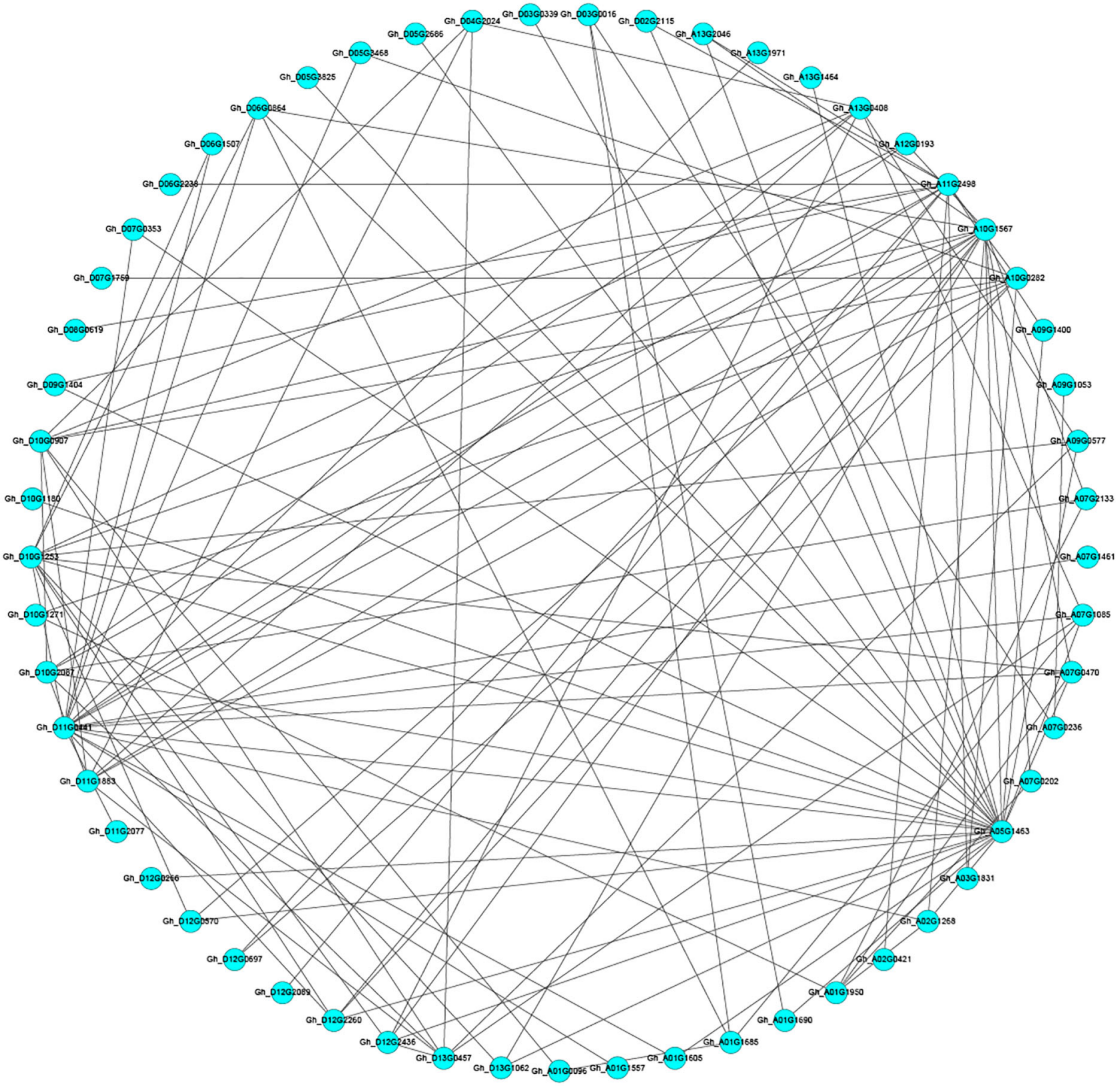
Analysis of predicted candidate gene protein interactions. Each circle in the figure represents a gene, and the lines between the circles represent interactions between different genes.

To further understand the regulatory mechanism of the candidate gene-encoded proteins under salt stress, the number of the candidate gene encoded protein was input into the string database to obtain the interaction relationship of the candidate gene encoded protein. The resulting interactions are visualized using cytoscape software (**Figure 2**). The bioinformatics prediction results of 16 candidate gene encoded proteins obtained 41 proteins interacting with them. In the image, the blue dots represent the candidate gene encoding protein, and the two dots with black lines indicate that the two proteins interact.

### qRT-PCR analysis of candidate genes

**Figure 3:**
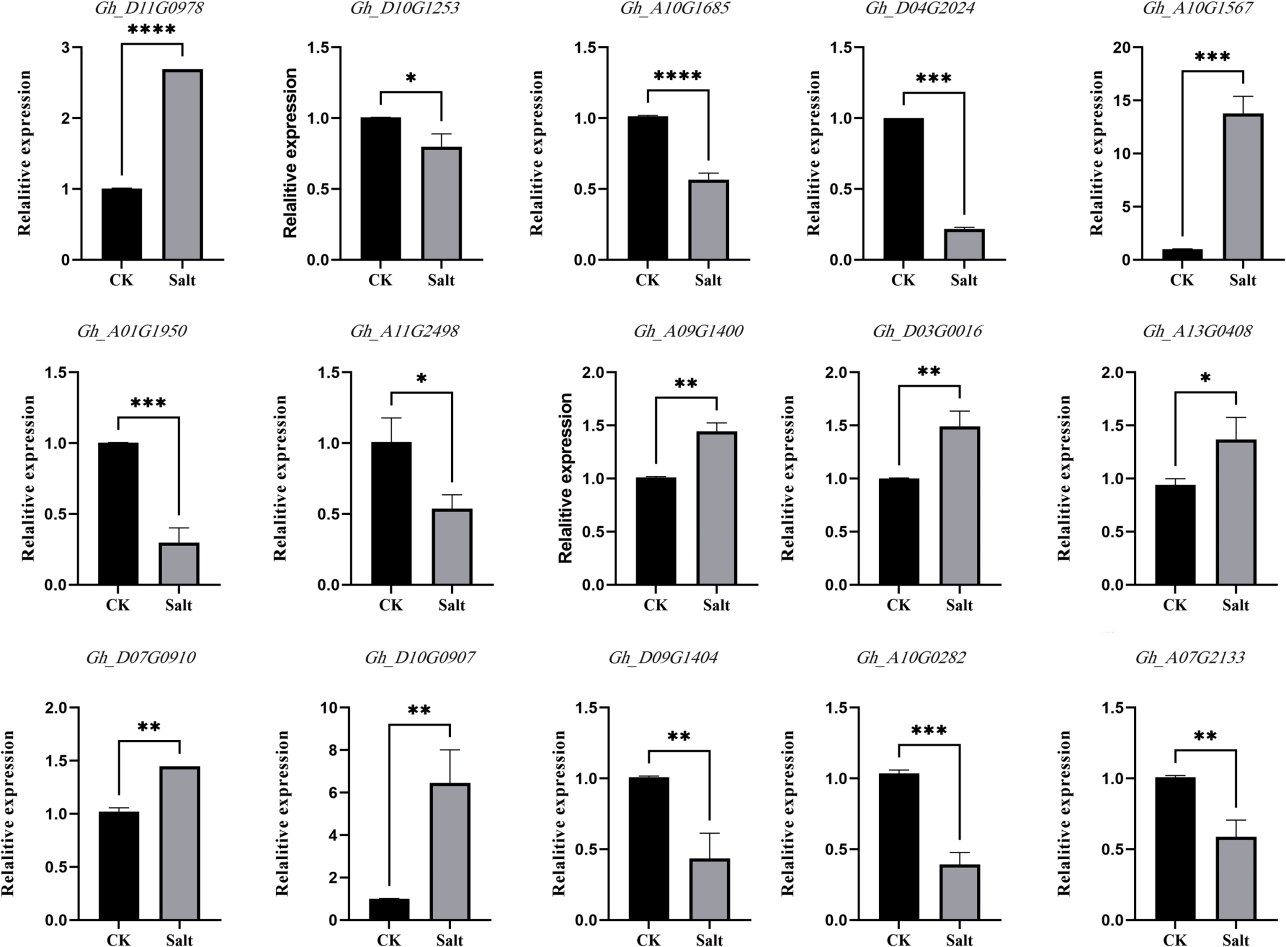
Candidate gene expression validation. The abscissa in the figure is the sample name, and the ordinate is the relative expression. (** means P ≤ 0.01 in T test, **** means P ≤ 0.0001 in T-test).

Among the 24 genes obtained from the integrated transcriptome and proteome analysis, 15 genes with large fold changes were selected for qRT-PCR experiments in this study. Specific primers for the candidate genes were designed using Primer5 software (**Table S1**), and the results are shown in **Figure 3**. The results of the qRT-PCR experiments were generally in agreement with the results of the transcriptome and proteome, and the expression trends of the DEGs were mostly consistent, thus verifying the accuracy of the transcriptome and proteome data.

### Functional verification of two candidate genes by VIGS under salt stress

**Figure 4:**
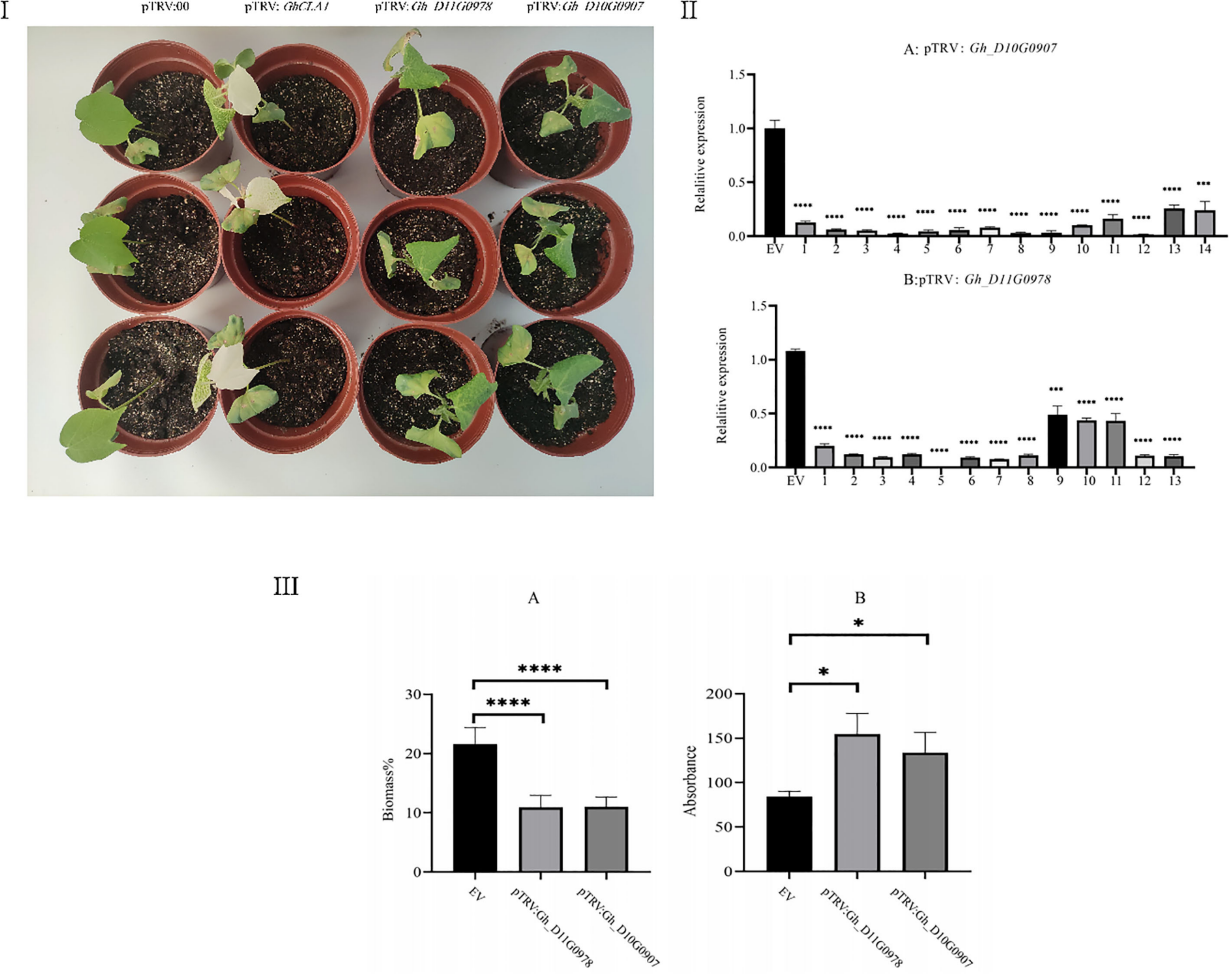
**I**: Phenotype of VIGS plants after 7 d of salt treatment. In the figure from left to right are pTRV:00 (negative control, empty vector), pTRV : *GhCLA1* (positive control, silencing the *GhCLA1* gene), pTRV : *Gh_D11G0978* (silencing the *Gh_D11G0978* gene), and pTRV : *Gh_D10G0907* (silencing the *Gh_D10G0907* gene). **II**: **(A)**: Gene expression of pTRV : *Gh_D10G0907*-silenced cotton. **(B)**: Gene expression of pTRV : *Gh_D11G0978*-silenced cotton. **III**: **(A)**: Comparison of the biomass ratio of roots and stems in the negative control, pTRV : *Gh_D10G0907*, and pTRV : *Gh_D11G0978*. **(B)**: Comparison of the ROS content of leaves in the negative control, pTRV : *Gh_D10G0907*, and pTRV : *Gh_D11G0978*.

To verify the functions of two significant DEGs in the regulation of salt stress (*Gh_D11G0978* and *Gh_D10G0907*) obtained from integrated transcriptomic and proteomic analysis, upland cotton plants in which these two genes were silenced were created by VIGS. After 10 days of VIGS treatment, bleaching of the true leaves occurred in the positive control plants, implying that the *GhCLA1* gene was successfully silenced (**Figure 4I**); qRT-PCR analysis suggested that the expression levels of the target genes *Gh_D10G0907* and *Gh_D11G0978* were significantly decreased compared to the negative control after 10 days of VIGS treatment (**Figure 4II**). These results indicated that our VIGS procedure is correct and effective (Zhang et al., 2017).

Compared with the negative control with a slightly wilted phenotype, the silenced plants of the genes *Gh_D10G0907* and *Gh_D11G0978* were subjected to retarded development, severe wilting, and dry symptoms on the leaf margins and yellow spots on the leaves (**Figure 4**). In addition, both silenced plants had significantly lower relative biomass and higher ROS content than the negative control (**Figure 4III**). These results suggest that the genes *Gh_D10G0907* and *Gh_D11G0978* play an important role in the pathway of cotton response to salt stress. They have a positive impact on enhancing salt stress tolerance in upland cotton.

## Discussion

Based on transcriptome and proteome analysis, a total of 10,967 DEGs and 189 DEPs were obtained respectively from salt-tolerant upland cotton ‘Tong Yan No. 1’, and then 24 candidate genes were obtained from the integrated analysis of DEGs and DEPs. The qRT-PCR validation of these candidate genes revealed significant differential expression in upland cotton after salt treatment. The present study therefore suggests that these 24 candidate genes have an important role in salt tolerance in upland cotton.

GO annotation of candidate genes showed that upland cotton plants had a relatively strong stress response to salt stress and that the internal structure and metabolic processes of the plants were damaged to some extent. KEGG analysis of candidate genes also showed that salt stress significantly enriched the production of antioxidant substances, terpene biosynthesis, membrane lipid metabolism and pathways associated with antioxidant adversity. Under salt stress, upland cotton reduces cell damage by producing antioxidant substances to scavenge ROS, regulating intracellular, physiological and biochemical responses and ensuring the normal functioning of the photosynthetic and respiratory systems (Zhang et al., 2016a). The accelerated metabolism of lipids ensures the integrity of the cell membrane, helps to maintain the normal morphology of the cells, and ensures the normal transport of substances (Yang and Guo, 2018a). In addition, the accelerated metabolism of sugars maintains cell wall morphology, protects the cell structure, and participates in the regulation of osmotic pressure (Fan et al., 2015; Guo et al., 2015; Zhang et al., 2016b). Hormone synthesis, such as abscisic acid (ABA), is also an important method for cotton to resist salt stress (Wang et al., 2010). The bioinformatics analysis of candidate gene encoded proteins and the prediction of proteins interacting with candidate gene encoded proteins can provide a comprehensive understanding of the function and regulatory mechanism of candidate genes. The binding interactions of Gh_A01G1605, a gene encoding ethanol dehydrogenase (ADH1), and *Gh_D11G0441*, a gene encoding acetaldehyde dehydrogenase (ALDH10A8) have been reported (Missihoun et al., 2015). Homologs of these two genes were associated with salt stress in rice, with significant changes in expression under salt stress (Liang et al., 2012). These two enzymes regulate metabolic processes in plant tissues. Under different concentrations of NaCl stress, there were significant changes in the expression of ADH3 and ALDH in rice, as ethanol dehydrogenase protects the structural and functional integrity of membranes when rice is subjected to salt stress, while ALDH catalyzes the oxidation of aldehydes to carboxylic acids, which removes toxic aldehydes, reduces lipid peroxidation and improves salt tolerance in plants.

This study identified many salt tolerance genes through the combined analysis of the transcriptome and proteome in upland cotton. These genes are involved in different physiological and biochemical pathways, but they all respond significantly to salt stress and reduce salt damage in different pathways. Under salt stress, cotton plant cells are severely damaged by osmotic pressure, and the genes that synthesize sugars and osmotic substances have obvious expression changes to maintain the balance of osmotic pressure inside and outside cells (Acosta-Motos et al., 2017). *Gh_D11G0978*, homologous to the *LEA14-A* gene in *A. thaliana*, which is associated with embryonic development and involved in the regulation of osmosis, has been found to be upregulated in response to different stresses (Jia et al., 2014). Overexpression of *LEA14* in *A. thaliana* and sweet potato increased tolerance to dehydration and salt stress. Overexpression of *LEA14* in sweet potato embryonic healing tissues increases tolerance to salt stress through enhanced lignification (Park et al., 2010). In addition, *OsLEA5* in rice enhances resistance to a variety of abiotic stresses (Miyazaki et al., 2021). Overall, LEA proteins are closely associated with resistance to a variety of abiotic stresses and can increase plant resistance to drought and salt tolerance.

Under salt stress, the cell membrane of cotton plants is seriously damaged (Abdelraheem et al., 2019). To maintain cell shape and internal and external material transport, many lipid synthesis- and cell membrane synthesis-related genes also had significant differential expression (Yang and Guo, 2018a). *Gh_D10G0907* encodes a glycerol glucoside phosphate synthase (GGPS), which can catalyze the synthesis of glycerol-3-phosphate and ADP-glucose into glycerol glucose-phosphate to maintain cell membrane structure. When plants are exposed to salt stress, the synthesis of glycerol glucoside begins in a two-step reaction to reduce salt stress (Takashima et al., 2020; Klähn et al., 2021). Phosphatidylcholine (PC) is not only an important component of cell membranes but also a source of signaling molecules. The *Gh_A01G1685* gene encodes serine decarboxylase (SDC)-catalyzed PC biosynthesis, converting serine to ethanolamine. Choline/ethanolamine kinase catalyzes the initial reaction step of choline metabolism to produce phosphoethanolamine. In leaves, the upregulated expression of SDC may be associated with cell membrane synthesis and the synthesis of betaine and phosphatidic acid (PA) (Li et al., 2020; Zhang et al., 2021). *Gh_A01G1950* encodes sulfoquinoline transferase 2 (SQD2) and is related to the sulfoquinoline diacylglycerol (SQDG) biosynthetic pathway. *SQD2.1* in rice has a dual role in catalyzing SQDG synthesis and flavonoid glycosylation. The mutants and overexpression lines of *SQD2.1* in rice exhibited reduced and enhanced tolerance to salt stress, respectively (Zhan et al., 2019). *Gh_A07G2133* encodes glycerol phosphodiester phosphodiesterase (GDPD), a family of evolutionarily conserved hydrolases that catabolize a range of glycerol phosphodiesters to sn-glycerol-3-phosphate and alcohol. The relative expression of the gene was shown to be significantly downregulated in barley roots when the plants were subjected to salt stress (Sarabia et al., 2020). In the upland cotton salt stress transcriptome study, *GDPD* gene expression was also significantly downregulated after salt stress.

ABA is a hormone known to be involved in plant stress resistance (Ullah et al., 2018). The ABA pathway is triggered to participate in the regulation of osmotic stress when the salt concentration increases. After salt treatment, the ABA synthesis speed and concentration increased, and the enhanced ABA signal activated sucrose nonfermenting 1-related protein kinase 2 (SnRK2). SnRK2 phosphorylates various ABA response elements and transcription factors that further regulate stomatal closure in plants in response to osmotic stress. Under salt stress, ABA-activated SnRK2 can also participate in amylolysis to regulate osmotic homeostasis. The regulation of stomata by ABA is an important strategy for plants to cope with NaCl-induced osmotic stress (Yu et al., 2020; Wu et al., 2021). There are two pathways of ABA synthesis: direct ABA synthesis by FPP or ABA synthesis by cleavage of carotenoids (Cutler and Krochko, 1999; Finkelstein, 2013; Lotfi et al., 2022). In this study, four candidate genes, *Gh_A13G0408*, *Gh_D10G0907*, *Gh_D11G1883* and *Gh_D13G0457*, were found to be enriched in the terpenoid backbone biosynthesis pathway, which belongs to the largest group of secondary metabolites according to the KEGG enrichment analysis; *Gh_D11G1883* and *Gh_D10G0907* both encode GGPS. There are two biosynthetic pathways for terpenoid backbone biosynthesis: the mevalonate pathway, which produces mevalonate, and the nonmevalonate pathway, which produces geranyl pyrophosphate (GPP), farnesyl pyrophosphate (FPP) and geranylgeranyl diphosphate (GGPP). These products in the nonmevalonate pathway are condensed to produce sterol (C30) and carotenoid (C40) precursors (Takashima et al., 2020; Klähn et al., 2021). The expression levels of the four candidate genes were all significantly upregulated under salt stress. Therefore, we speculate that the four genes are involved in the regulation of the terpenoid backbone biosynthesis pathway, including the synthesis of FPP and carotenoid precursors, thus affecting the ABA synthesis pathway. Both *Gh_D10G0907* and *Gh_D13G0457*, two copies of the *ISPH* gene, were significantly upregulated in expression, which might accelerate the synthesis of ABA precursors and promote ABA synthesis to resist salt stress (Wang et al., 2010). *Gh_D11G1883* and *Gh_D10G0907* are two copies of the *GGPS* gene, but they have different expression patterns under salt stress, which can be explained by functional redundancy.

VIGS is an effective method to verify gene function and has been used in many plants (Jin et al., 2016). In this study, the role of two important candidate genes, *Gh_D10G0907* and *Gh_D11G0978*, verified by integrated transcriptome and proteome analysis under salt stress was further identified by VIGS. The gene-silenced plants displayed significant phenotypic changes in leaf wilting, drying and biomass compared to the negative control, which is consistent with the phenotype of silencing plants for the salt tolerance gene *GhEXO70B1* (Zhang et al., 2016; Ding et al., 2018). The gene-silenced plants were significantly less tolerant to salt stress than the negative control. Therefore, the target genes *Gh_D10G0907* and *Gh_D11G0978* played an important role in the salt tolerance pathway in upland cotton. Further study of their functional mechanism will provide a basis for salt-tolerant cotton breeding. Since the silenced plants were less tolerant to salt stress, the damage to the cell structure was also more severe. The photosynthetic system is severely damaged, and a large number of nutrients are broken down in the plant for respiration, resulting in a lower relative biomass in the gene-silenced plants than in the negative control. A large amount of bound water leaked out after the cell structure was disrupted, and the plants themselves were more easily dried out, with a higher water loss rate in silent plants at the same temperature and time, consistent with the findings of biomass changes under salt stress treatments of the plants (Guo et al., 2015). The downregulation of the expression of the target gene was demonstrated to reduce the salt tolerance of cotton (Gao et al., 2011). Under salt stress, cotton produces a large amount of ROS, and the higher the level of ROS is, the greater the damage caused to the cotton plant. The presence of large numbers of ionic free radicals changes the internal biochemical environment of cotton, preventing normal physiological and biochemical reactions and accelerating the aging of the cotton plant, as well as damaging the mitochondria and chloroplasts inside the cells. The high levels of ROS were evidence of cotton plants that were more severely damaged by salt stress, and the silenced plants had higher levels of ROS than the negative control, indicating that the silenced plants were subjected to more severe salt stress than the control plants (Zhang et al., 2016a; Wei et al., 2017).

## Conclusion

Cotton is known as a salt-tolerant crop, and the mechanisms of salt tolerance have yet to be identified. In this study, many DEGs and DEPs were obtained through the joint analysis of transcriptome and proteome sequencing under salt stress. We therefore narrowed down the candidate genes by focusing our study on genes with significant differences and consistent expression trends. Two genes were identified to participate in the response to salt stress in upland cotton. The integrated analysis of the transcriptome and proteome of upland cotton under salt stress will help to uncover high-quality salt tolerance genes and provide a basis for the study of salt stress response pathways in cotton.

## Data availability statement

The original contributions presented in the study are publicly available. The data presented in the study are deposited in the NGDC repository, accession number PRJCA011934 (proteome) and PRJCA011990 (transcriptome).

## Author contributions

KS, TGM, and HF performed most of the experiments and data analysis. JH, XH, JXZ, YC, DW, and ZZ helped in sample preparation. AD, MK and JZ helped design the experiments and revise the manuscript. KW and BW designed the experiments and edited the manuscript. All authors contributed to the article and approved the submitted version.

## Supplementary material

Supplementary Figure 1 Annotation statistics of GO secondary nodes for differentially expressed genes. The horizontal coordinate is the GO classification, the left side of the vertical axis is the percentage of the number of genes, and the right side is the number of genes.

Supplementary Figure 2 Results of GO enrichment analysis. The vertical coordinate is the enriched GO term, the horizontal coordinate is the enriched q value, and the numbers on the bars are the number of differential genes. The different colors represent biological processes and cellular components.

Supplementary Figure 3 Graph of KEGG enrichment statistics for differentially expressed genes. The vertical axis indicates the pathway name, the horizontal axis indicates the rich factor, the size of the dots indicates the number of DEGs, and the color of the dots corresponds to the q-value.

Supplementary Figure 4 Annotation statistics of GO secondary nodes for differentially expressed proteins. The horizontal coordinate is the GO classification, the left side of the vertical axis is the percentage of the number of genes, and the right side is the number of genes.

Supplementary Figure 5 Results of GO enrichment of differentially expressed proteins. The vertical coordinate is the enriched GO term, the horizontal coordinate is the enriched q value, and the numbers on the bars are the number of differential genes. The different colors represent biological processes and cellular components.

Supplementary Figure 6 Plot of statistical results of differential protein KEGG enrichment. The vertical axis indicates the name, the horizontal axis indicates the Rich factor, the size of the dots indicates the number of differentially expressed proteins, and the color of the dots corresponds to the different q-value ranges.
