# Supplementary figures and images for: Transcriptome, proteome and functional characterization reveals salt stress tolerance mechanisms in upland cotton (Gossypium hirsutum L.)
Source: Front Plant Sci. 2023 Feb 16;14:1092616. doi: 10.3389/fpls.2023.1092616 (PMC9978342; doi:10.3389/fpls.2023.1092616)

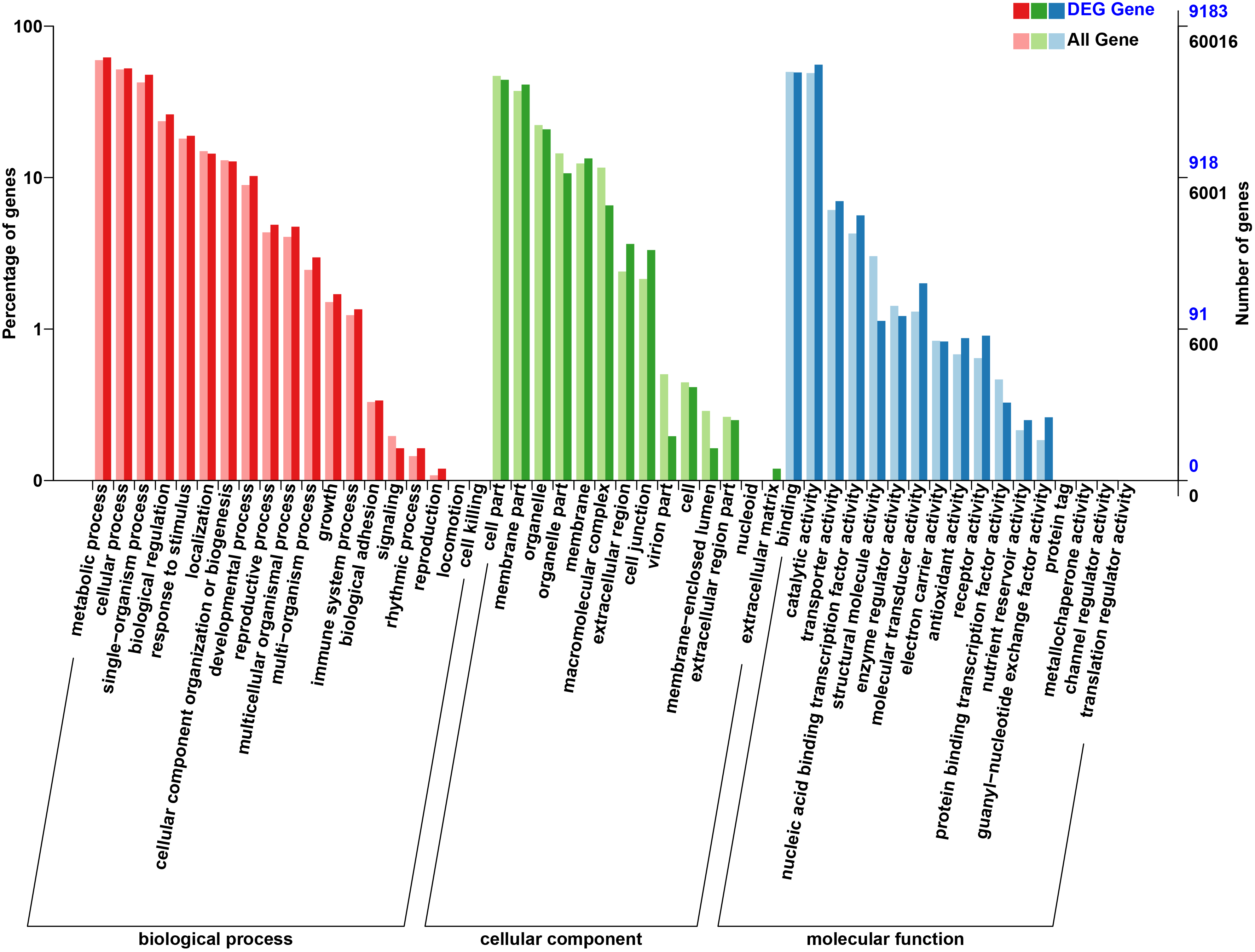

Supplement: Supplementary Figure 1 — Annotation statistics of GO secondary nodes for differentially expressed genes. The horizontal coordinate is the GO classification, the left side of the vertical axis is the percentage of the number of genes, and the right side is the number of genes. [file Image_1.tif]

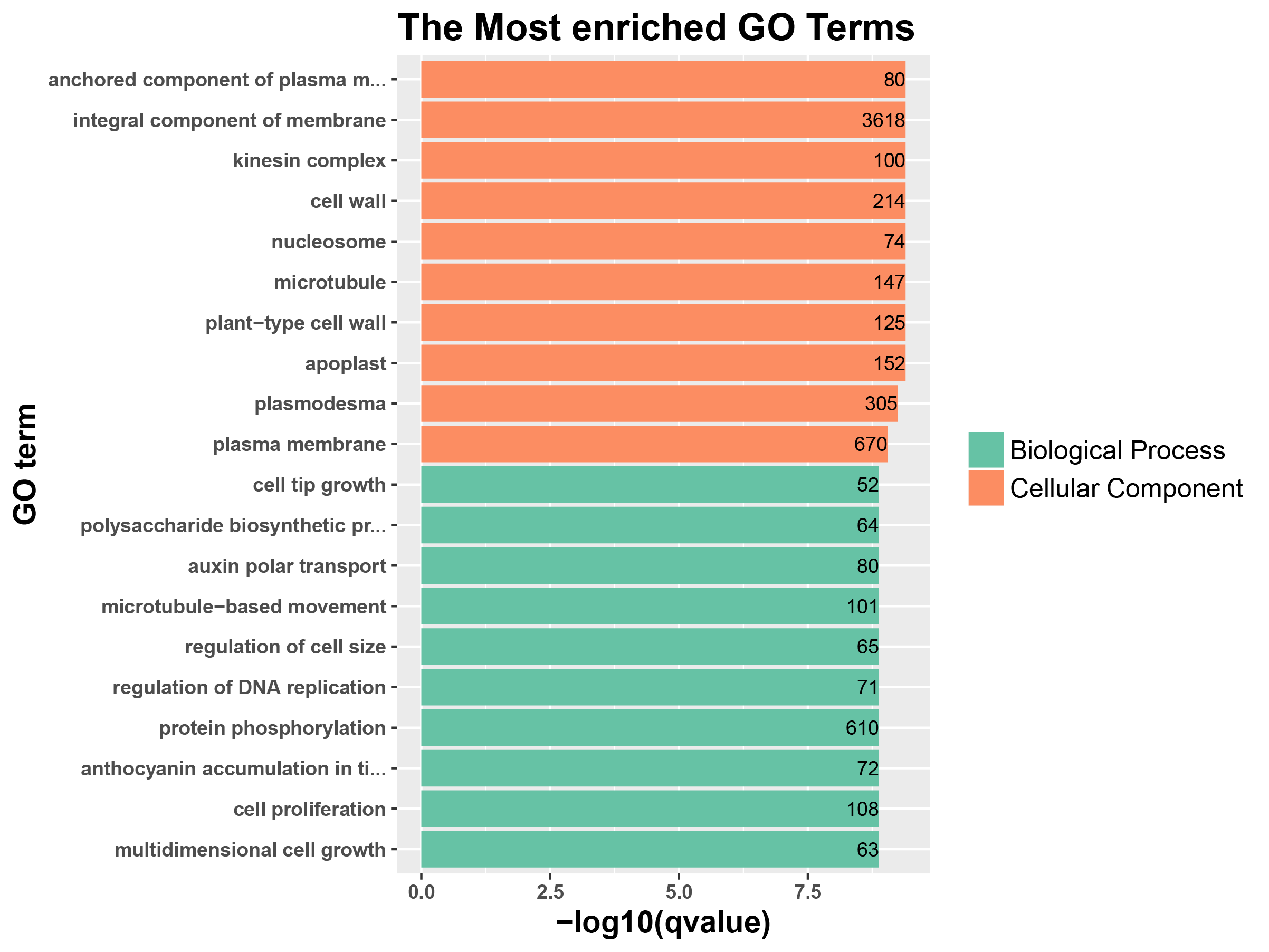

Supplement: Supplementary Figure 2 — Results of GO enrichment analysis. The vertical coordinate is the enriched GO term, the horizontal coordinate is the enriched q value, and the numbers on the bars are the number of differential genes. The different colors represent biological processes and cellular components. [file Image_2.tif]

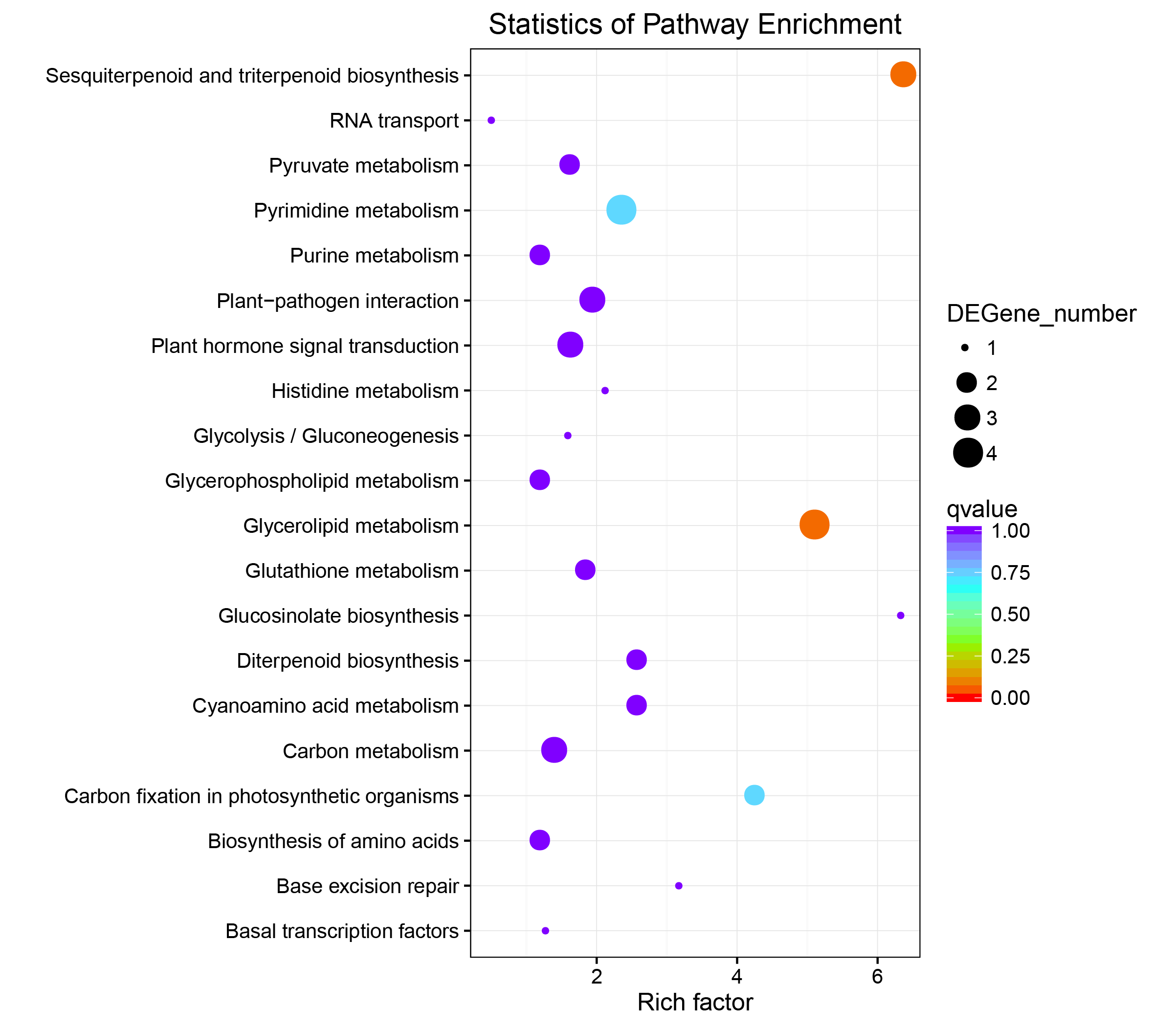

Supplement: Supplementary Figure 3 — Graph of KEGG enrichment statistics for differentially expressed genes. The vertical axis indicates the pathway name, the horizontal axis indicates the rich factor, the size of the dots indicates the number of DEGs, and the color of the dots corresponds to the q-value. [file Image_3.tif]

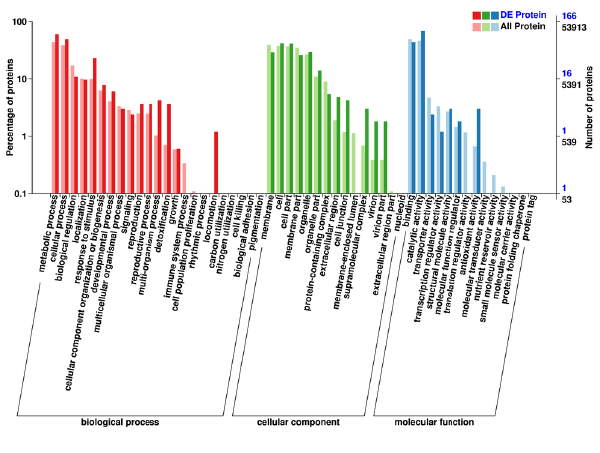

Supplement: Supplementary Figure 4 — Annotation statistics of GO secondary nodes for differentially expressed proteins. The horizontal coordinate is the GO classification, the left side of the vertical axis is the percentage of the number of genes, and the right side is the number of genes. [file Image_4.tif]

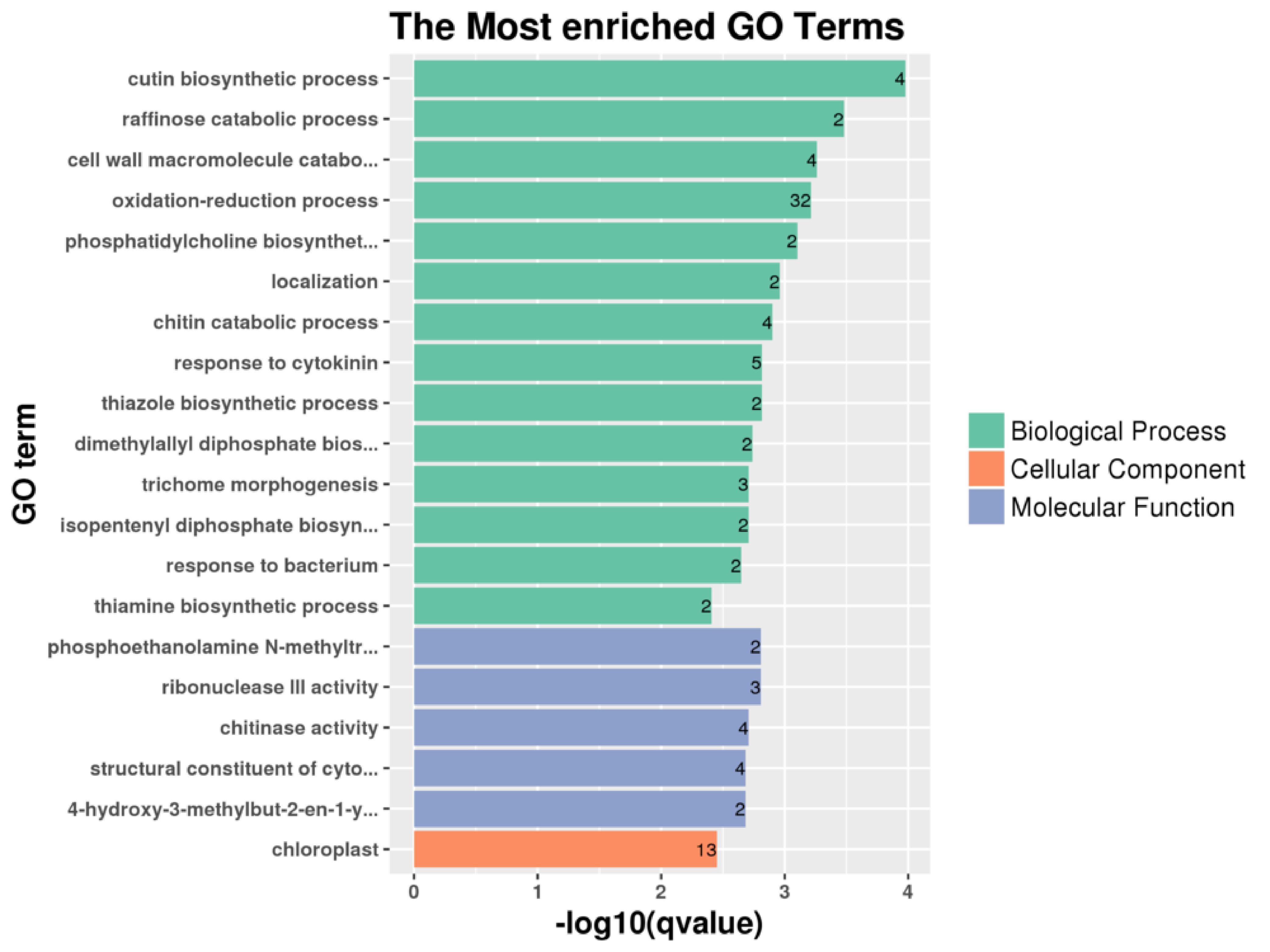

Supplement: Supplementary Figure 5 — Results of GO enrichment of differentially expressed proteins. The vertical coordinate is the enriched GO term, the horizontal coordinate is the enriched q value, and the numbers on the bars are the number of differential genes. The different colors represent biological processes and cellular components. [file Image_5.tif]

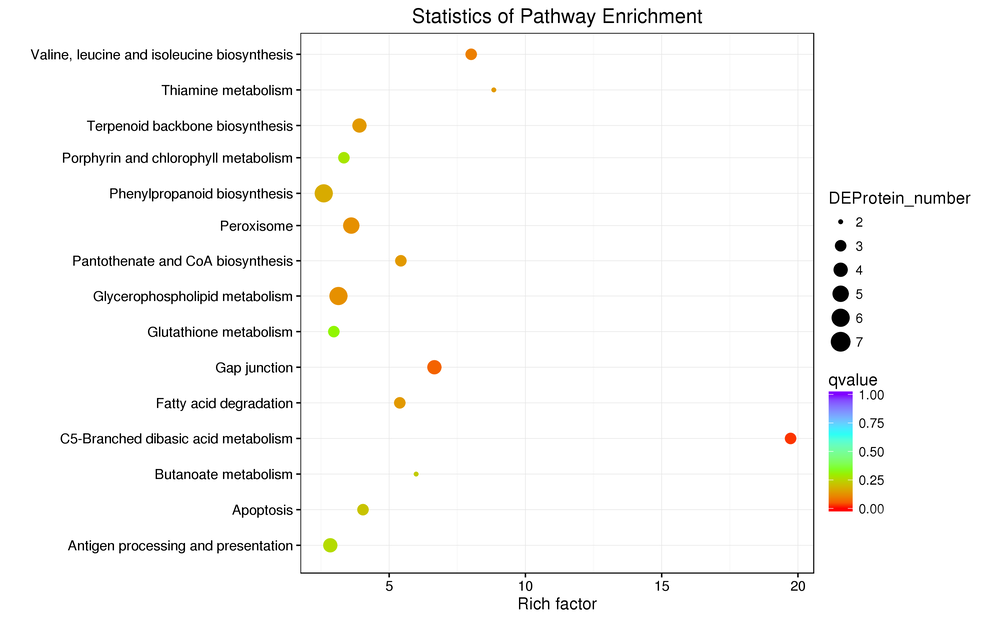

Supplement: Supplementary Figure 6 — Plot of statistical results of differential protein KEGG enrichment. The vertical axis indicates the name, the horizontal axis indicates the Rich factor, the size of the dots indicates the number of differentially expressed proteins, and the color of the dots corresponds to the different q-value ranges. [file Image_6.tif]
